# Utilization of Electronic Devices and Online Education Tools for Learning Among Undergraduate Medical Students of a Tertiary Care Teaching Hospital: A Cross-Sectional Study

**DOI:** 10.7759/cureus.90862

**Published:** 2025-08-24

**Authors:** Tejas A Acharya, Chaitanya G Chinawale, Ruchita J Mer, Sunita B Chhaiya, Madhav D Trivedi

**Affiliations:** 1 Department of Pharmacology, C. U. Shah Medical College and Hospital, Surendranagar, IND; 2 Department of Physiology, C.U. Shah Medical College and Hospital, Surendranagar, IND

**Keywords:** cross-sectional study, digital learning platforms, electronic devices, online education tools, undergraduate medical students

## Abstract

Introduction

Mobile learning and digital educational tools have revolutionized medical education globally. This study explores the usage patterns and perceptions of electronic devices and online educational tools among undergraduate medical students in a tertiary care hospital in India.

Materials and methods

A cross-sectional descriptive study was conducted at a tertiary care teaching hospital among undergraduate medical students. A validated questionnaire covering three domains (general use, learning use, and attitudes) was distributed through Google Forms (Google LLC, Mountain View, CA, USA). Data were analyzed for statistical association of the usefulness of online educational tools and the attitude of students toward online education tools by the chi-square test. Cronbach's alpha was calculated for attitude questions.

Results

Of 400 students, 322 (80.5%) participated. Smartphones were the most commonly used devices among 151 (46.9%) students, and 252 (78.26%) students used online educational platforms. Marrow and YouTube were the most popular educational platforms. Most students found online tools helpful in various aspects. For learning new concepts, 319 (99.1%) students; for revision, 297 (92.2%) students; and for self-assessment, 288 (89.4%) students stated online tools as helpful, which was statistically significant (p<0.05). While students appreciated digital learning, many remained neutral or skeptical about replacing traditional methods. Association for all the questions of attitude was statistically significant (p<0.05).

Conclusions

Undergraduate medical students at our tertiary care teaching hospital widely adopt digital education tools. While digital platforms enhance learning, challenges such as cost and potential distractions warrant consideration in overemphasizing the use of online education tools.

## Introduction

The integration of electronic devices and digital learning platforms into medical education has undergone a transformative shift over the past decade. The widespread availability of smartphones, tablets, and laptops has enabled students to access vast amounts of medical content on demand, fostering self-directed and flexible learning environments. This evolution has been particularly evident with the rise of mobile learning (m-learning), which leverages portable technology to support education beyond traditional classroom boundaries.

Recent studies have shown that m-learning not only enhances knowledge acquisition but also improves clinical skill development. A meta-analysis by Liu et al. reported a significant effect of mobile learning on educational outcomes in health profession education, especially when integrated with structured pedagogical strategies [1]. In a similar vein, Schlenz et al. found that digital learning platforms, including mobile applications and video-based modules, are perceived as more engaging and efficient than traditional lecture formats among medical students [2].

The COVID-19 pandemic acted as a catalyst, accelerating the global adoption of online education within medical curricula. Medical institutions rapidly transitioned to virtual classrooms, simulation-based e-learning, and tele-teaching platforms to continue delivering education despite lockdowns. A systematic review by Pei et al. demonstrated that digital teaching modalities could produce clinical outcomes and examination performance comparable to in-person instruction, while also improving cost-effectiveness and learner satisfaction [3]. However, disparities in access to high-speed internet, suitable devices, and digital literacy emerged as significant barriers, particularly in low- and middle-income countries [4].

Moreover, the growing use of augmented reality (AR) and virtual reality (VR) technologies in medical education has introduced new dimensions to anatomical and clinical instruction. Recent reviews highlight their potential in improving spatial understanding and learner engagement, especially when combined with interactive 3D models and virtual patient cases [5].

Despite these advancements, there remains a gap in understanding the real-world use, preferences, and perceptions of digital educational tools among medical students in specific institutional contexts. In particular, few studies have examined how undergraduate students in Indian medical colleges, especially those in tier-two cities or resource-limited settings, utilize digital tools for both general and academic purposes. Such insights are critical for curriculum planning, infrastructure development, and targeted faculty training.

Therefore, this study was conducted with an overall aim to assess the utilization of electronic devices and online educational tools for learning among undergraduate medical students in a tertiary care teaching hospital in India. The objectives of the study were to determine the use of electronic devices, the use of online educational tools, and the perceived attitude toward using online educational tools among undergraduate medical students.

## Materials and methods

Ethical consideration

The study was carried out at a tertiary care teaching hospital after obtaining written permission from the Institutional Ethics Committee (Human Research) of C. U. Shah Medical College, Surendranagar (approval number: USMC/IEC(HR)/RP/26/2024/Fina Approval/212/2024).

Study design and setting

It was a cross-sectional descriptive study conducted on all undergraduate medical students of a tertiary care teaching hospital in India.

Inclusion criteria

All undergraduate medical students who were willing to give consent.

Sample size determination

As the study targeted the entire student population of the institute, no prior sample size calculation was done. The response rate justified the adequacy of the collected data.

Instrument development

A questionnaire was prepared with reference to previously conducted similar studies [6-8]. A few modifications were made, and new questions were added. The questionnaire consists of three sections and 20 questions. One section was regarding the general use of electronic devices and had five questions. The second section focused on learning the use of electronic devices and consisted of nine questions. The third and final section, which assessed the attitude of students, had a total of six questions. Four external faculties further validated the prepared questions. A content validity index was calculated both at the item level (I-CVI) and scale level (S-CVI) [9]. The faculty members were asked to rate each item based on relevance, clarity, simplicity, and ambiguity on a four-point scale. Content validity was measured by a four-point content validity index (CVI). The content validity of the questionnaire was assessed using the S-CVI/Ave method. The I-CVIs ranged from 0.75 to one, and S-CVI/Ave was calculated to be 0.98 to one, indicating a high level of content validity. Finally, prepared questions are listed in Table 1.

**Table 1:** Validated questionnaire for evaluation of online education PC: personal computer

General use of electronic devices
Which is your favourite device for online learning? Smartphone, laptop/PC, tablet
Do you have access to an internet connection with proper speed? Yes, no
Do you have a smartphone? Yes, no
Which type of smartphone do you have? Android, iPhone, Windows, BlackBerry
Which is your most frequently used activity on your smartphone? Online education, social media, gaming, surfing
Learning to use electronic devices
Do you use any online education application or platform for learning medical subjects? Yes, no
Which online education application or platform is most valuable for you in learning subjects? (Open-ended)
How frequently do you use your electronic device for learning? Daily, weekly, fortnightly, monthly
Are any social media platforms useful for learning medical subjects? Yes, no
Which social media platform is most valuable in learning medical subjects? YouTube, WhatsApp, Facebook, Twitter
Which is your most frequently used smartphone activity for learning? Watching lecture videos, surfing text material, communicating with departments, taking online assessments
Are online educational tools helpful in learning new concepts? Yes, no
Are online educational tools helpful in the revision of subjects? Yes, no
Are online educational tools helpful in self-assessment? Yes, no
Attitude of students (strongly disagree/disagree/neutral/agree/strongly agree)
Online education tools improve access to learning material.
Electronic devices help to learn independently.
Smart phone can be a distracter in learning process.
Online educational tools can totally substitute conventional teaching methods.
Use of smartphone for teaching can be beneficial to faculties.
Online education tools are much expensive.

The questionnaire was converted into a Google Form (Google LLC, Mountain View, CA, USA). The Google Form was subdivided into seven sections. Section one was a participant information sheet with details of the title and nature of the study, along with details of the investigators. Section two was informed consent, where students can agree or decline to participate. If the student agrees to participate, then only the form moves to section three, which is about demographic details. Sections four to six were proper questionnaires as mentioned in Table 1, and lastly, section seven contained a thank-you note to the participants. All the questions were compulsory to prevent incomplete or missing data.

Participant recruitment and data collection

All the undergraduate medical students (phases 1, 2, and 3) were included phase-wise in the study. First, there are phase 1 students, followed by phase 2 students, and then phase 3 students. Data collection for all students was completed within 15 days. The investigator visited the class of each phase for data collection. Recruitment was carried out in the classroom settings at the end of scheduled lectures, with prior permission of the faculty. Before sharing the Google Form, students were provided with enough instructions regarding the nature of the study. Students who were willing to give informed consent, which was part of the Google Form, were included in the study. Absent students were approached during subsequent sessions to maximize coverage.

Statistical analysis

All the responses were compiled in a Microsoft Excel (Microsoft Corp., Redmond, WA, USA) sheet and analyzed using descriptive and inferential statistics. Categorical variables (gender, device type, platform used, etc.) were summarized as frequencies and percentages. The association between perceived usefulness and the independent variable of online educational tools was established using the chi-square test. For Likert scale responses of the attitude of the students, data were presented as proportions across the categories. To further evaluate the association between students' attitudes toward online educational tools, the chi-square test was applied. For both, p<0.05 was considered statistically significant. The Cronbach’s alpha was also calculated to establish the reliability and internal consistency of the items about the attitude of students toward online educational tools. The value of Cronbach’s alpha between 0.7 and 1 was considered as acceptable to excellent internal consistency. All the analyses were performed in Microsoft Excel 2016, with confirmatory tests run in SPSS Statistics version 24 (IBM Corp. Released 2016. IBM SPSS Statistics for Windows, Version 22.0. Armonk, NY: IBM Corp.).

## Results

Demographic distribution

Out of 400 students across all phases, 322 students responded to questions. The achieved responses (N=322, 80.5%) provide a high response rate, ensuring adequate representativeness of the study population. Among the study population, 94 (29.19%) students were from the first MBBS, which was the highest. Out of 322 students, female students outnumbered male students. The ages of students ranged from 17 to 24 years. The maximum age of students was 20 years old. Demographic distribution of students is summarized in Table 2.

**Table 2:** Demographic distribution Data mentioned in the table represent the number of students (N) and the percentage (%). MBBS: Bachelor of Medicine, Bachelor of Surgery

Category	Sub-category	Number of students (N=322)
Phase-wise distribution	First MBBS	94 (29.19%)
	Second MBBS	89 (27.63%)
	Third MBBS part 1	63 (19.56%)
	Third MBBS part 2	76 (23.60%)
Gender distribution	Female	172 (53.41%)
	Male	150 (46.58%)
Age-wise distribution	17	8 (2.48%)
	18	47 (14.6%)
	19	62 (19.25%)
	20	77 (23.91%)
	21	65 (20.18%)
	22	42 (13.04%)
	23	14 (4.35%)
	24	7 (2.17%)

General use of electronic devices

The smartphone was identified as the most preferred device for online learning, as indicated by 151 (46.90%) students. 269 (83.54%) of participant students reported having access to a stable internet connection with proper speed. Nearly all students have a smartphone, with a substantial majority utilizing Android-based devices. Responses to questions regarding the general use of electronic devices are briefed in Table 3.

**Table 3:** General use of electronic devices Data mentioned in the table represent the number of students (N) and the percentage (%). PC: personal computer

Sr. No.	Question	Options	Number (N=322)
1	Which is your favourite device for online learning?	Tablet	132 (40.99%)
		Laptop/PC	39 (12.11%)
		Smartphone	151 (46.90%)
2	Do you have access to an internet connection with proper speed?	Yes	269 (83.54%)
		No	53 (16.46%)
3	Do you have a smartphone?	Yes	320 (99.38%)
		No	2 (0.62%)
4	Which type of smartphone do you have?	Android	292 (90.63%)
		iPhone	30 (9.38%)

**Figure 1:**
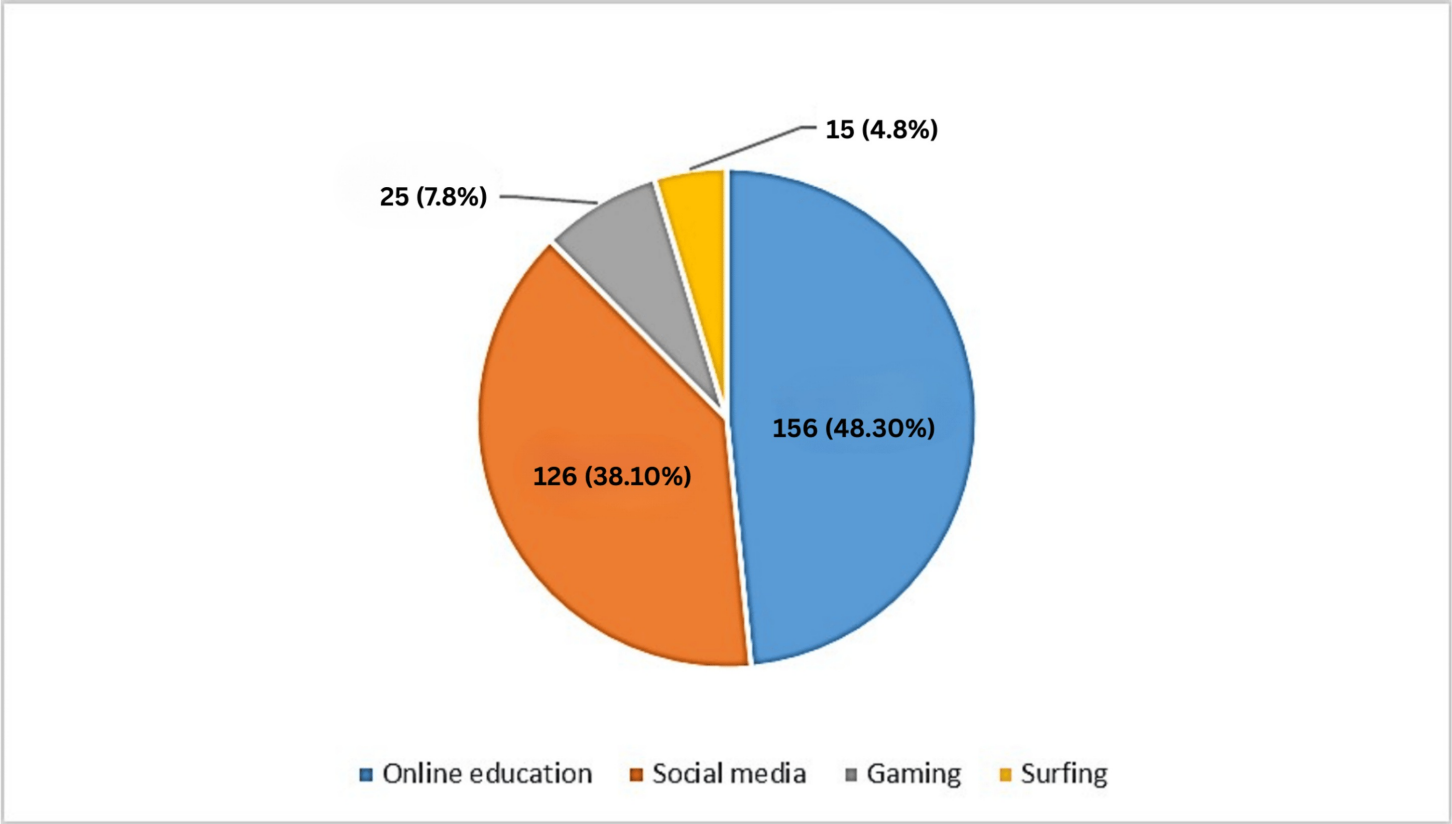
Most frequent activities on smartphones by students (N=322) Data mentioned in the figure represent the number of students (N) and the percentage (%).

The most common activities done by students on smartphones are depicted in Figure 1, which shows online education as the most common, with 156 (48.30%) students, followed by the use of social media, with 126 (38.10%) students performing on smartphones.

Learning to use electronic devices

**Figure 2:**
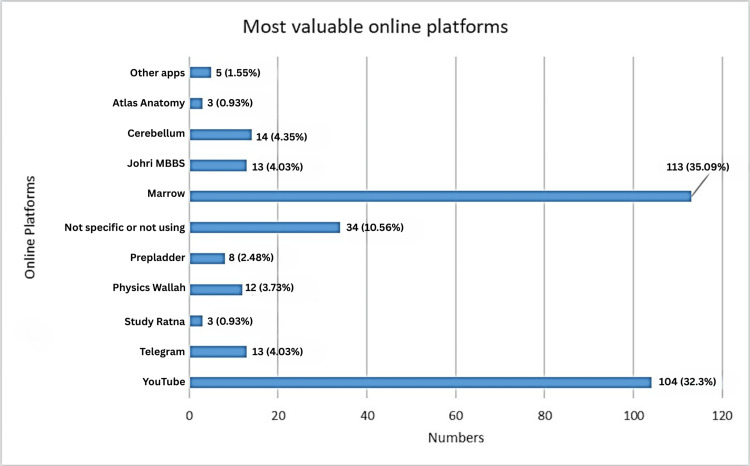
Most helpful online platforms for medical education Data mentioned in the figure represent the number of students (N) and the percentage (%). MBBS: Bachelor of Medicine, Bachelor of Surgery

Out of 322 students, 252 (78.26%) students reported using online education applications or platforms for learning medical subjects. Details of various platforms used by students are represented in Figure 2. Marrow was the most frequently used platform among 113 (35.09%) students, followed by YouTube among 104 (32.3%) students. Marrow was most commonly used by third MBBS part-two students, with usage among 59 (52%) students, while YouTube was the favorite among first MBBS students, with usage among 33 (45%) students.

Regarding frequency of device usage, 263 (81.68%) students reported that they use it daily for online learning; 294 (91.30%) students agreed that social media platforms are also helpful in learning medical education, among which YouTube, with 303 (94.10%) students, was the most valuable social media platform for learning. Watching video lectures online, with usage among 288 (89.44%) students, was the most common activity. Responses to questions about learning from the use of electronic devices are illustrated in Table 4.

**Table 4:** Learning use of electronic devices Data mentioned in the table represent the number of students (N) and the percentage (%).

Sr. No.	Question	Options	Number (N=322)
1.	Use of an online education platform for medical subjects	Yes	252 (78.26%)
		No	70 (21.74%)
2.	Frequency of electronic device use for learning	Daily	263 (81.68%)
		Weekly	49 (15.22%)
		Fortnightly	5 (1.55%)
		Monthly	5 (1.55%)
3.	Usefulness of social media in learning medical subjects	Yes	294 (91.30%)
		No	28 (8.70%)
4.	Most valuable social media platform for medical learning	YouTube	303 (94.10%)
		WhatsApp	15 (4.66%)
		Twitter	3 (0.93%)
		Facebook	1 (0.31%)
5.	Most frequently used smartphone activity for learning	Watching lecture videos	288 (89.44%)
		Surfing text material	21 (6.52%)
		Communication with departments	7 (2.17%)
		Online assessment	6 (1.86%)

The response regarding the usefulness of educational tools among students is described in Table 5. They were convinced about the usefulness of online educational tools in aspects like learning, revision, and self-assessment. To establish an association of the responses, the chi-square test was applied, which revealed that it was statistically significant (p<0.05).

**Table 5:** Usefulness of online educational tools Data mentioned in the table represent the number of students (N) and the percentage (%). * X^2^ value denotes value of chi-square test, ** p<0.05 is kept at a statistically significant level

Question	Yes	No	Statistical test	p-value**
Are online educational tools helpful in learning new concepts?	319 (99.1%)	3 (0.9%)	X^2^*=310.11	p<0.0001
Are online educational tools helpful in the revision of subjects?	297 (92.2%)	25 (7.8%)	X^2^*=229.76	p<0.0001
Are online educational tools helpful in self-assessment?	288 (89.4%)	34 (10.6%)	X^2^*=200.36	p<0.0001

Attitude of students

The attitude of students toward the usefulness and cautions about online educational tools and platforms is described in Table 6.

**Table 6:** Attitude of students toward online educational tools Data mentioned in the table represent the number of students (N) and the percentage (%). * X^2^ value denotes value of chi-square test,** p<0.05 is kept at a statistically significant level

Attitude questions	Strongly disagree	Disagree	Neutral	Agree	Strongly agree	Statistical test	p-value**
Online education tools improve access to learning materials	16 (5%)	7 (2.2%)	65 (20.2%)	159 (49.4%)	75 (23.2%)	X^2^*=228.24	p<0.0001
Electronic devices help to learn independently	15 (4.7%)	13 (4%)	64 (19.9%)	158 (49.1%)	72 (22.3%)	X^2^*=215.85	p<0.0001
Smartphone can be a distracter in the learning process	17 (5.3%)	22 (6.8%)	110 (34.2%)	117 (36.3%)	56 (17.4%)	X^2^*=139.14	p<0.0001
Online educational tools can totally substitute conventional teaching methods	26 (8.1%)	49 (15.2%)	139 (43.2%)	87 (27%)	21 (6.5%)	X^2^*=150.17	p<0.0001
Use of smartphones for teaching can be beneficial to faculty	9 (2.8%)	18 (5.6%)	108 (33.5%)	155 (48.2%)	32 (9.9%)	X^2^*=254.36	p<0.0001
Online education tools are much expensive	13 (4%)	21 (6.5%)	120 (37.3%)	117 (36.4%)	51 (15.8%)	X^2^*=164.02	p<0.0001

It is evident from the table that students have mixed types of attitudes toward online learning. They agree with the usefulness of online teaching aids. At the same time, they are neutral about replacing conventional teaching methods entirely and cautious about the expense of online teaching tools. On applying the chi-square test, the association between responses for each question was found statistically significant (p<0.05). Cronbach's alpha for questions on the attitude of students was 0.727, which reflects an acceptable level of internal consistency, implying that questions demonstrate a satisfactory degree of reliability in assessing the intended construct.

## Discussion

The present study highlights the widespread integration of electronic devices and online educational tools among undergraduate medical students in a tertiary care teaching hospital in India. With 320 (99.38%) of students reporting smartphone ownership and over 252 (78.26%) using online educational platforms for academic purposes, the findings resonate with global trends in digital learning within medical education.

These results align with a 2021 meta-analysis by Liu et al., which demonstrated that mobile learning (m-learning) improves both knowledge acquisition and skill development compared to traditional methods, particularly when devices like smartphones and tablets are actively used in instruction and in a blended manner [1]. In our study, smartphones were the most frequently used devices for online learning among 151 (46.90%) students, followed by tablets among 132 (40.99%) students. This reflects a growing trend wherein mobile-first access dominates student learning habits, especially in low- and middle-income settings.

The majority of students, with a significant number of 263 (81.68%), reported using devices daily for learning, and 303 (94.10%) students identified YouTube as a key educational resource. These findings are supported by Alsoufi et al., who observed that YouTube and similar platforms were widely accessed during the COVID-19 pandemic for both didactic and clinical teaching [10]. The use of online learning platforms and social media has also survived the COVID-19 pandemic.

Students also overwhelmingly acknowledged the usefulness of digital tools: 319 (99.1%) students found them helpful in learning new concepts, 297 (92.2%) students for revision, and 288 (89.4%) students for self-assessment. These results echo a 2019 systematic review and meta-analysis by Pei et al., which found that online learning has its own advantages and should be considered as a potential source for stimulating students' learning [3].

However, students’ attitudes toward online tools replacing conventional teaching were more cautious: only 21 (6.5%) students strongly agreed with the substitution, while 139 (43.2%) students remained neutral. This cautious optimism is reflected in other studies, such as a 2021 cross-sectional analysis by Dost et al., which found that while students appreciated the flexibility of online learning, many were skeptical about its ability to fully replicate in-person experiences, especially in clinical contexts [11].

Interestingly, students also acknowledged potential downsides. For instance, 173 (53.7%) students agreed or strongly agreed that smartphones can be distracting during learning. This concern is consistent with findings from Wilcha, who noted that digital tools, while beneficial, can contribute to decreased focus due to multitasking and notification overload [12].

Another important point is the economic concern: 168 (52.2%) students perceived online education tools to be expensive. However, this is partially supported by the review done by O’Doherty et al. [4], which suggested that from an institutional perspective, online education may be more cost-effective than traditional methods. Still, at the student level, cost remains a matter of concern.

Despite the above, a positive outlook was noted on the potential for smartphones to aid faculty: 187 (58.1%) students agreed or strongly agreed that smartphones can support teaching. This supports current literature on faculty adoption of mobile-enhanced pedagogies, which improves flexibility in content delivery and communication [13].

Finally, the real-world implications of this study are significant. As digital health technologies, such as VR, AR, and tele-simulation, become increasingly prominent, foundational digital fluency among undergraduate students becomes critical. In the future, advanced technologies may completely transform the learning and teaching process for medical students and teachers, respectively. Emerging evidence, like the review by Maresky et al., supports the efficacy of AR/VR in improving anatomical understanding and engagement [5].

Limitations

The current study was restricted to a single tertiary care teaching hospital with a relatively smaller sample size, and results may not be generalized. Larger studies including multiple centers are required for further clarification and establishment of long-term effectiveness. Although all efforts were made, including a proper explanation by the investigator, an anonymous reply, the use of neutral language, and data collection in a controlled environment, there are still chances of response bias.

## Conclusions

This was an observational study to analyze the trend of utilizing online learning in medical education. Based on the results, the use of electronic devices is very prevalent at our tertiary care teaching hospital, which represents the current generation of medical students. Online education is a common use of electronic devices at our institute. Marrow and YouTube were the most commonly used online education platforms in our study population. Students showed a positive attitude toward the usefulness of online education tools in all aspects of learning. At the same time, they were neutral regarding the replacement of the conventional education system and also cautious about the price of online educational tools, which should warrant consideration in overemphasizing the usage of online education tools.
